# Pediatric Text Neck Syndrome

**DOI:** 10.7759/cureus.38034

**Published:** 2023-04-23

**Authors:** Eden YT Chu, Tze Kwan Sharon Mok, Gabriel Siu Nam Ng, Eric Chun-Pu Chu

**Affiliations:** 1 Chiropractic and Physiotherapy Centre, New York Medical Group, Hong Kong, CHN

**Keywords:** cervicogenic dyspnea, cervicogenic vertigo, cervicogenic dizziness, chiropractor, chiropractic, cervicogenic headache, text neck

## Abstract

Text neck syndrome is a growing concern in the pediatric population due to the increased use of mobile devices and screens, potentially leading to long-lasting musculoskeletal issues. This case report presents a six-year-old boy with a one-month history of cephalgia and cervicalgia, who initially received insufficient care. After nine months of chiropractic intervention, the patient reported significant improvements in pain relief, neck mobility, and neurological symptoms, supported by radiographic findings. This report emphasizes the importance of early recognition and intervention in pediatric patients, as well as the role of ergonomics, exercise, and proper smartphone usage habits in preventing text neck and maintaining spinal health.

## Introduction

Text neck syndrome is an overuse syndrome typically caused by repetitive stress injury of the cervical spine due to flexed and forward head position during prolonged engagement with mobile screens [1]. If not adequately addressed, the pain and other symptoms of the cervical spine can progressively worsen, leading to a range of physical health issues such as spinal disc degeneration, musculoskeletal pain, and reversed cervical lordosis [2]. Cervical spine disorders include cervicogenic headaches [3], cervicogenic dizziness [2], cervicogenic angina and dyspnea [4,5], facial pain [6], fibromyalgia [7], visual disturbance [8,9], and cervical radiculopathy [10,11], which often present with a combination of painful symptoms and spinal dysfunction.

The pediatric population is particularly vulnerable to the development of text necks due to the increasing use of mobile devices and screens at a young age [2]. Prolonged exposure to poor postural habits in children may result in long-lasting musculoskeletal issues and a higher risk of spine-related disorders later in life [2]. Chiropractors are educated to recognize structural or movement abnormalities that could cause injury or discomfort if left untreated [12,13]. They focus on the early detection and repair of musculoskeletal dysfunction to prevent progression to more serious disorders [14,15]. Therefore, early recognition and intervention for text neck syndrome in children are essential for addressing neck problems and preventing further complications.

## Case presentation

A six-year-old boy presented with a one-month history of headache, cephalgia, and cervicalgia. The insidious onset of symptoms coincided with prolonged usage of a mobile device (more than eight hours per day), primarily for viewing videos. The patient described the pain as a constant, non-pulsatile discomfort localized to the posterior and inferior aspects of the right cranium. The pain occasionally radiated to the right cervical region and interscapular area and was sometimes accompanied by ipsilateral frontal and supraorbital involvement.

The patient’s pain was quantified as 7 on a numeric pain rating scale ranging from 0 to 10. The World Health Organization’s Quality of Life assessment indicated an 80% quality of life score for the patient. The episodes typically persisted for a minimum of 30 minutes but sometimes extended over several days. The patient reported that maintaining a flexed posture, particularly while engaging in handheld gaming using his mobile device, exacerbated the symptoms. In contrast, hydrotherapy, such as hot showers and baths, temporarily alleviated the discomfort. The patient’s daily screen time exceeded 10 hours, including gaming and video-watching activities. Approximately 48 hours after the initial manifestation of symptoms, the patient consulted a pediatrician. The prescribed management plan included non-steroidal anti-inflammatory drugs (e.g., aspirin or ibuprofen), muscle relaxants, and a regimen of stretching and exercise. However, these interventions failed to provide significant relief. Subsequently, the patient sought chiropractic care to address his condition.

**Figure 1:**
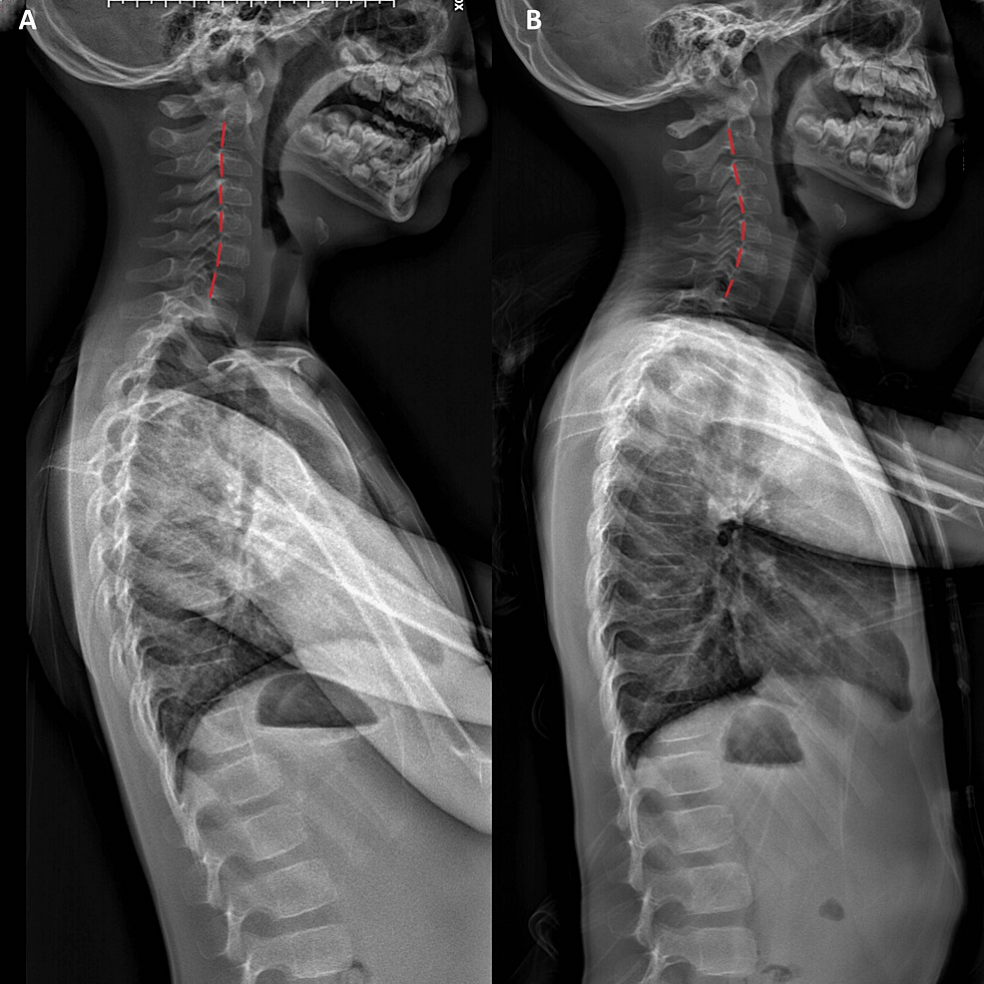
EOS® (EOS Imaging, Paris, France) full-spine radiography in sagittal view. (A) Radiographic imaging of the cervical spine revealed a reduction in cervical lordosis (red line) before treatment. (B) At the nine-month follow-up, the patient’s cervical range of motion was improved bilaterally and cervical lordosis was restored (red line).

**Figure 2:**
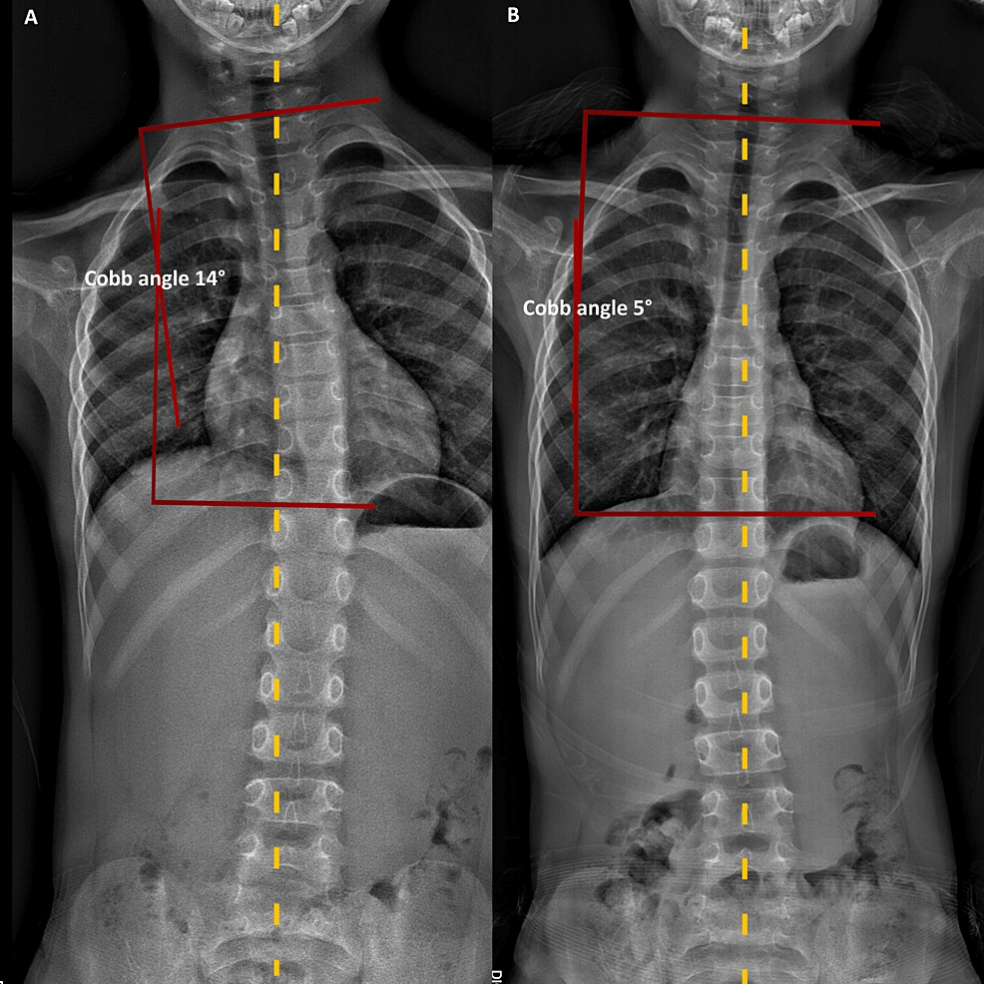
EOS® full-spine radiography in frontal view. (A) Shoulder asymmetry and thoracic scoliosis were present before chiropractic intervention. The central sacral vertical line (yellow line) signifies the global axis. (B) At the nine-month reassessment, the patient showed notable improvements in postural balance. The radiographic parameter showed improvement of Cobb angle measurement from 14° (to the left) to 5° (to the right), and assessment of the central sacral vertical line (yellow line) revealed a considerable improvement in the coronal balance of spinal scoliosis.

The patient presented to a chiropractor with kyphotic posture, scapular asymmetry, and lateral cervical flexion. Upon palpation, tenderness was noted on the right side of the scalp and the cervical and shoulder muscles. Muscular hypertonicity was identified in the rectus capitis, rectus longus, upper trapezius, serratus anterior, and levator scapulae. Intersegmental dysfunction was palpated at the C5-6, T1-2, and T7-8 vertebral levels. An orthopedic evaluation revealed a limited range of motion in the cervical spine, specifically 40° of right rotation (normal >80°) and 25° of extension (normal >50°). The Adam test indicated scoliosis. A comprehensive neurological examination assessing cognitive function, cranial nerves, reflexes, sensory system, motor system, cerebellar function, and gait revealed no abnormalities. EOS® (EOS Imaging, Paris, France), a low-dose biplanar digital radiographic imaging system, demonstrated loss of cervical lordosis and thoracolumbar scoliosis (Figure 1, Panel A and Figure 2, Panel A). Based on the patient’s clinical presentation and radiological findings, text neck syndrome with associated cervicogenic headache was initially diagnosed. Chiropractic manipulation was used to re-establish postural stability and correct spinal alignment by comparing pre and post-treatment radiography.

The therapeutic approach for patients with text neck syndrome incorporates a combination of spinal adjustment techniques, cervical extension-traction therapy, and instrument-assisted soft tissue manipulation, with the primary objectives of rectifying shoulder asymmetry, spinal misalignment, and muscle tension. Spinal adjustment techniques were employed at the spinal segments exhibiting intersegmental dysfunction to restore the alignment of the spine. Cervical extension-traction therapy, utilizing the iTrac® Spine Remodeling System, aims to re-establish normal cervical curvature. An instrument-assisted soft tissue manipulation tool (Strig, Seoul, Korea) was used to alleviate muscle tension and promote spinal stability. In the first month, the patient underwent 12 therapy sessions. Following this period, the patient’s pain rating on the numeric pain scale decreased from 7 to 0. Additional recommendations, such as optimizing ergonomic advice on mobile device use, reducing screen time, and incorporating core strengthening exercises, were provided to address the patient’s forward head posture and excessive kyphosis.

For the subsequent two months, the frequency of therapeutic visits was decreased to eight per month. By the end of the third month, the patient reported a complete resolution of symptoms. To manage the patient’s residual spinal imbalances, therapy continued at a frequency of four times per month. At the nine-month follow-up, the patient remained headache-free, and his cervical range of motion was restored bilaterally. The assessment indicated a 100% quality of life score for the patient. Moreover, substantial improvements were observed in radiographic parameters, including cervical curvature restoration and correction of thoracic spine dextroconvexity (Figure 1, Panel B and Figure 2, Panel B).

## Discussion

The increasing prevalence of social media, mobile texting, video streaming, and gaming due to the widespread adoption of smartphones has led to emerging health concerns, including the overuse condition known as text neck syndrome [16-18]. As mobile device usage continues to increase, the pediatric population is becoming particularly vulnerable to the development of kyphotic deformities in the growing and premature spinal structure [2]. When a child first develops their spine, the upper cervical segments serve as the fulcrum for cervical flexion [19].

Pediatric anatomical variants that do not lead to a significant reduction of physical function and disability can be considered congenital and physiological anomalies [20]. However, repetitive mechanical stress applied to these minor variants can exacerbate biomechanical alterations, predisposing the cervical spine to segmental instability [2]. Over time, sustained flexed stress may cause posterior ligamentous laxity, vertebral segment instability, spondylolisthesis, and degenerative joint disease [2,21], possibly leading to plastic impairment within the nervous system and causing clinical symptoms [21]. This underscores the importance of early recognition and intervention in pediatric patients to prevent long-term complications.

Even 10 minutes of static spinal flexion position can alter the neuromuscular and mechanical signals of the cervical structure [22]. Therefore, monitoring the pediatric population for the development of text neck syndrome and related issues is crucial. Although conservative treatments such as manual therapy have demonstrated clinical benefits in correcting reversed cervical curvature [2,7,23,24], interventions should be tailored to the unique needs of children, taking into account their developing anatomy, growth patterns, and biomechanics for addressing text neck syndrome. In the present case, extension traction therapy aimed at the anterior longitudinal ligament was used, assuming that the restoration of natural cervical lordosis was primarily due to ligamentous creep (stretching) [25]. In addition, educational efforts should be directed toward promoting proper smartphone usage habits and ergonomic adjustments among children to minimize the risk of developing text neck and other spinal health problems. Furthermore, regular exercise and core strengthening programs should be encouraged to promote healthy spines [26] and mitigate the potential adverse effects of excessive smartphone use on pediatric spinal health.

## Conclusions

This report highlights the progression of insufficiently treated text neck and the consequences of sustained neck flexion on cervical spine distortion. The observed improvements in cephalgia and cervicalgia correlated with the radiographic changes in response to cervical misalignment correction. These findings emphasize the importance of early recognition and intervention for text neck in the pediatric population, the potential benefits and limitations of chiropractic treatments, pediatric patient considerations, and the role of ergonomics and exercise in maintaining spinal health. Although the study did not explore the potential contributing factors, such as lifestyle, genetics, or environmental influences, which could have an impact on the patient’s condition and response to treatment, preventing text neck through increased awareness and proper smartphone usage habits is crucial for mitigating preventable repetitive injuries.
